# Exploring the Causal Relationship Between Modifiable Exposures and Diabetes Mellitus: A Two-Sample Mendelian Randomization Analysis

**DOI:** 10.7759/cureus.59034

**Published:** 2024-04-25

**Authors:** Mohammed A Muaddi

**Affiliations:** 1 Family and Community Medicine Department, Jazan University, Jazan, SAU

**Keywords:** obesity, lifestyle, dietary habits, diet, smoking, mendelian randomization, diabetes, modifiable risk factors

## Abstract

Background

Observational studies link lifestyle factors to diabetes, but confounding limits causal inference. This study employed Mendelian randomization (MR) to investigate the potential causal effects of major dietary, obesity, smoking, and physical activity exposures on diabetes risk.

Methods

A two-sample MR framework integrated FinnGen and United Kingdom Biobank (UKB) data. Genetic instruments for diet (fruits, vegetables, cheese), smoking (initiation, intensity, maternal), body mass index (BMI), and physical activity came from various consortia (n=64, 949-632, 802). Associations with diabetes odds were assessed using inverse-variance weighted analysis.

Results

Fruit and cheese intake and physical activity per standard deviation increase causally reduced diabetes risk in both cohorts. Conversely, smoking initiation, maternal smoking around birth, and BMI per standard deviation increase causally increased diabetes risk in both cohorts. Coffee increased diabetes risk only in FinnGen, whereas smoking intensity increased diabetes risk only in UKB.

Conclusion

This study provides robust evidence that modifiable lifestyle factors may have causal effects on diabetes risk. Fruit, cheese, and physical activity may protect against diabetes, whereas smoking, maternal smoking, and higher BMI appear to increase risk. Findings support public health interventions targeting diet, physical activity, smoking cessation, and healthy weight to combat the global diabetes epidemic.

## Introduction

Diabetes mellitus (DM) has emerged as one of the most prevalent metabolic diseases worldwide, with rising incidence rates that are of serious global concern [1,2]. Over the past few decades, there has been a dramatic four-fold increase in the rates of type 2 diabetes worldwide. Nearly 700 million people are projected to have diabetes by 2045 [3]. This rise has coincided with growing trends in obesity, physical inactivity, and aging populations between 1980 and 2004 [4]. With diabetes now the sixth leading cause of disability globally as of 2015 [5], this disease exerts immense burdens on individuals, families, communities, and health systems [6]. The socioeconomic impacts and costs associated with managing diabetes and its complications have become major concerns in the context of global health. Given the scale of this crisis, understanding modifiable risk factors and employing targeted prevention strategies are global health priorities.

Observational studies have linked several key lifestyle factors to increased diabetes risk, including obesity, dietary patterns, physical inactivity, and smoking [7-9]. However, these studies are limited in determining causation due to possible residual confounding inherent in observational analyses [10]. Other related socioeconomic, behavioral, or environmental factors may influence lifestyle habits and diabetes outcomes [11-13]. Experimental studies that can infer causal relationships are needed to specifically guide diabetes prevention policies, such as randomized controlled trials (RCTs). Manipulating dietary patterns or physical activity levels could provide higher-quality evidence of causation. However, RCTs can be resource intensive, practically challenging to conduct [14], and ethically concerning by knowingly exposing participants to harmful risk factors like physical inactivity, smoking, or unhealthful diets for extended periods to assess long-term diabetes outcomes [15].

Mendelian randomization (MR) has emerged as a technique to elucidate causal associations using genetic variants as instrumental variables for modifiable exposures [11,12]. MR may strengthen causal inference and address limitations in observational studies like confounding and reverse causation [16,17]. Despite major advances in MR methodology over the past decade, this technique has limited applications to comprehensively explore the causal associations between lifestyle factors and diabetes risk to date. In particular, MR studies evaluating key dietary components, including fruits, vegetables like salad, dairy products like cheese, caffeinated beverages like coffee, tobacco use patterns like smoking initiation and intensity, maternal smoking, obesity measured by body mass index (BMI), and physical activity levels are lacking, but are needed to inform diabetes prevention efforts.

Considering the substantial implications of elucidating these causal relationships for enhancing diabetes prevention and control, further, MR studies are critically needed to systematically and robustly interrogate the causal effects of these exposure factors on diabetes incidence in diverse populations. High-quality MR analyses can provide novel evidence to guide policies and interventions targeting dietary habits, smoking, obesity levels, and physical inactivity. Findings from such studies can validate the benefits of lifestyle and behavioral modifications for mitigating diabetes risk, thereby supporting the prioritization and implementation of such strategies for controlling the diabetes epidemic worldwide. The current study aims to employ a two-sample MR framework to investigate the potential causal effects of major lifestyle factors and dietary components on diabetes risk.

We hypothesize that factors like physical inactivity, smoking, and unhealthy dietary patterns will demonstrate causal effects on diabetes development. Findings can further validate lifestyle and dietary modification as strategies for diabetes prevention and control.

## Materials and methods

Mendelian randomization: A conceptual overview

**Figure 1:**
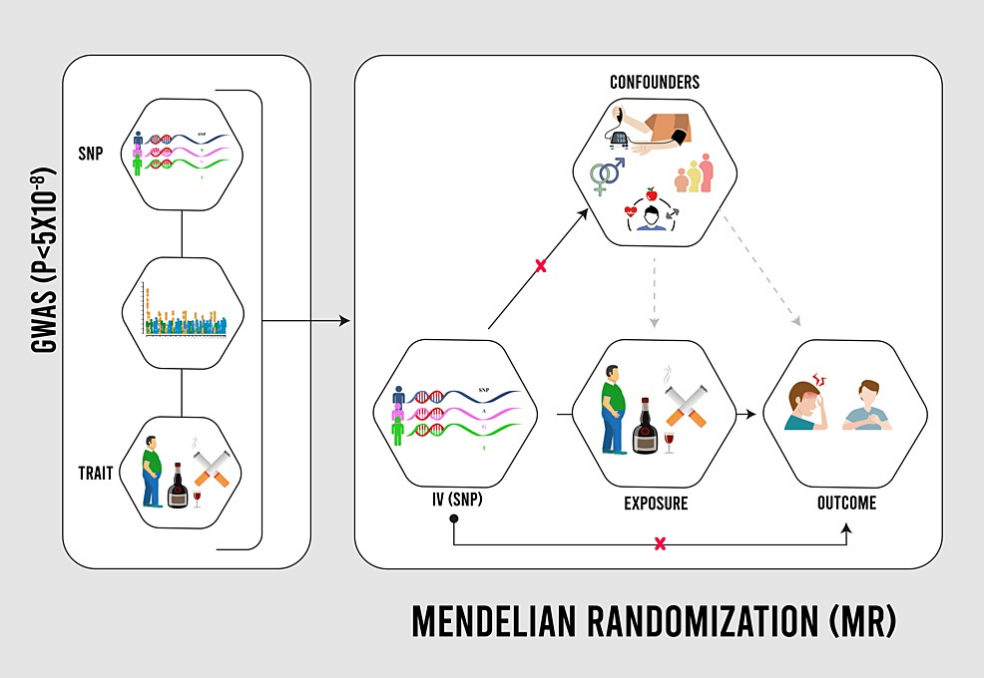
Conceptual diagram of Mendelian randomization methods Conceptual diagram of Mendelian randomization methods (adapted with permission from Jareebi MA. Understanding associations between smoking behaviour and poorer health: conventional and Mendelian randomization approaches. Doctoral thesis. University of Glasgow; (2022) [19] GWAS: Genome-Wide Association; SNPs: single nucleotide polymorphisms; IV: instrumental variable; MR: Mendelian randomization

MR is a statistical approach to quantifying causal associations between modifiable risk factors and outcomes using genetic variations of single nucleotide polymorphisms (SNPs) in the human genome [16]. SNPs, alterations in the DNA sequence, are identified through genome-wide association studies (GWAS), revealing significant associations with various traits (p-value <5x10^-8^) [18]. MR analysis uses these SNPs as proxies for risk factors, addressing limitations in observational studies such as confounding and reverse causation [17]. Assumptions include a significant SNP-risk factor association, no direct SNP-outcome relationship, and no SNP connection to confounding variables (Figure 1). MR can be one-sample (same population) or two-sample (different populations), requiring data from the same ancestry. This study adopts a two-sample MR to assess the causal relationship between risk factors and DM risk.

The UK Biobank and FinnGen cohorts

The UK Biobank (UKB) is a significant prospective cohort, including about 502,000 individuals assessed at 22 centers across England, Scotland, and Wales from 2006 to 2010. The evaluation covered diverse medical, psychosocial, and anthropometric parameters and self-reported and doctor-diagnosed medical conditions [20]. FinnGen, a genetic research initiative in Finland, aims to gather genetic data from 500,000 Finnish participants to explore associations between genetic factors and diseases. Starting in 2017 and expected to conclude in 2025, over 200,000 Finns have already contributed genetic data to support this extensive investigation [21]. This study relies on publicly accessible summary-level data from these integrated datasets. The current investigation delved into the potential causal role of dietary habits in DM development. Additionally, the study examined several potential risk factors that might impact DM risk, including fruit, salad, and cheese intake, coffee consumption, age at smoking initiation, smoking intensity, historical maternal smoking, BMI, and self-reported physical activity.

SNPs selection

We identified relevant variables by leveraging genome-wide significant SNPs from the following consortia: Pirastu et al. [22], the UKB [23], GWAS and Sequencing Consortium of Alcohol and Nicotine Use (GSCAN) [24], Genetic Investigation of Anthropometric Traits (GIANT) [25], and Klimentidis et al. [26]. These SNPs represent genetic variations associated with specific traits, as determined through GWAS at a significance threshold of p-value <5x10-8 [18]. The study employed a distinct set of SNPs, widely used in the literature [27,28], for various exposures. Specifically, there were 41 SNPs for fruit intake, 22 SNPs for salad intake, 65 SNPs for cheese intake, 3 SNPs for coffee consumption, 93 SNPs for smoking initiation, 23 SNPs for smoking intensity, 16 SNPs for maternal smoking, 79 SNPs for BMI, and 11 SNPs for physical activity.

Statistical analysis and integration of genetic data

Genetic information related to DM was sourced from two datasets: UKB and FinnGen [21]. Following data harmonization, involving the alignment and standardization of genetic associations, a set of SNPs for each exposure factor was examined in relation to DM. MR and sensitivity analyses were conducted using the TwoSampleMR package in R software (version 4.2.3). The analysis involved gathering genetic data for the exposures and their corresponding outcomes. Separate MR analyses were performed for DM using UKB and FinnGen consortia data. A significance level of P <0.05 was applied to all MR analyses, primarily focusing on the inverse variance weighted (IVW) method. Additionally, more stringent MR measures, including MR-Egger, accounting for increased pleiotropy, were employed to identify potential deviations from IVW results [16].

Data availability

The datasets analyzed in this study are publicly accessible to interested researchers through application to the respective cohort data access committees. The UK Biobank data can be requested by application to the UK Biobank data access committee [23], while access to FinnGen cohort data is available by application to the FinnGen Data Access Committee [21] after research proposals undergo evaluation and approval. The author accessed only summary-level data for this specific study through these public repositories without special access privileges beyond what any researcher could obtain through this standardized request process.

## Results

A total of 353 SNPs were examined across various risk factors, with the number of SNP variants assessed per factor ranging from 3 to 93. These genetic markers were obtained from different consortia with sample sizes varying between 64,949 and 632,802 individuals per risk factor (Table 1).

**Table 1:** Genetic Risk Factors in Brief: A Summary SNPs: single nucleotide polymorphisms; BMI: body mass index; UKB: UK Biobank; GWAS: Genome-Wide Association; GSCAN: GWAS & Sequencing Consortium of Alcohol and Nicotine Use; MRC-IEU: The Medical Research Council-Integrative Epidemiology Unit at the University of Bristol; GIANT: The Genetic Investigation of ANthropometric Traits

Exposure	No. of SNPs	Sample size	Population/consortium
Fruits	41	409,125	Pirastu et al. [22]
Salad	22	462,933	UKB
Cheese	65	451,486	UKB
Coffee	3	64,949	UKB
Smoking initiation	93	632,802	GSCAN
Smoking intensity	23	249,752	GSCAN
Maternal smoking	16	397,732	MRC-IEU [29]
BMI	79	339,152	GIANT
Number of days/weeks of vigorous physical activity	11	440,512	UKB

DM genetic characteristics

The genetic characteristics related to DM were investigated in two distinct population cohorts: the UKB and FinnGen. In the UKB cohort, comprising a total of 336,473 participants, 16,183 individuals were diagnosed with DM, as reported by medical professionals. Additionally, data from the FinnGen cohort, which consists of 215,654 participants, revealed that 32,469 individuals had been diagnosed with DM, while the remaining 183,185 served as controls in the analysis.

DM risk in the UKB cohort

The findings from the MR analysis evaluate potential causal relationships between lifestyle factors and risk of developing DM in the UKB consortium (Table 2). Examining dietary patterns, an 8% reduction in the risk of developing DM was associated with a genetically predicted higher intake of fruits (OR=0.92, 95% CI: 0.90-0.94, p<0.001). Similarly, a 4% decrease in DM risk was associated with genetically elevated cheese consumption (OR=0.96, 95% CI: 0.95-0.97, p<0.001). The impact of coffee consumption on DM risk was only marginally significant (OR=1.03, 95% CI: 0.99-1.07, p=0.09).

**Figure 2:**
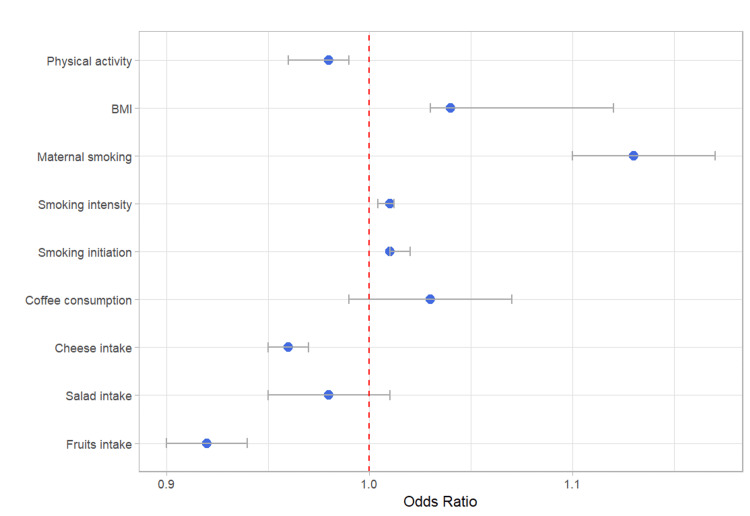
Dietary Habits and Lifestyle Impact on Diabetes Mellitus Risk: UK Biobank (UKB) Cohort. BMI: body mass index; UKB: UK Biobank

Lifestyle elements, including smoking initiation and intensity, demonstrated significant findings on DM risk. For smoking patterns, genetically estimated smoking initiation displayed a 1% increase in DM risk (OR=1.01, 95% CI: 1.01-1.02, p<0.001), genetically predicted greater smoking intensity demonstrated a significant relationship, with a 1% increase in DM risk per additional cigarette smoked per day (OR=1.01, 95% CI: 1.004 - 1.012, p<0.001). Genetically estimated maternal smoking history around birth showed a considerable impact, with a 13% increased risk of offspring developing DM (OR=1.13, 95% CI: 1.10-1.17, p<0.001) (Table 2, Figure 2).

Additionally, a higher body mass index (BMI) emerged as a considerable risk factor for DM, associated with a 4% increase in DM risk per one standard deviation higher BMI (OR=1.04, 95% CI: 1.03 - <0.001). Conversely, engaging in physical activity demonstrated a protective effect, manifesting as a 2% reduction in DM risk for genetically predicted physical activity (OR=0.98, 95% CI: 0.96-0.99, p=0.001) (Table 2, Figure 2).

**Table 2:** Overview of DM Findings in the UKB Cohort BMI: body mass index; CI: confidence interval; The asterisk indicates statistically significant values at p < 0.05 (*: P<0.05)

Risk factor	OR (95% CI)	P value
Fruits intake	0.92 (0.90 - 0.94)	<0.001*
Salad intake	0.98 (0.95 - 1.01)	0.169
Cheese intake	0.96 (0.95 - 0.97)	<0.001*
Coffee consumption	1.03 (0.99 - 1.07)	0.09
Smoking initiation	1.01 (1.01 - 1.02)	<0.001*
Smoking intensity	1.01 (1.004 - 1.012)	<0.001*
Maternal smoking	1.13 (1.10 - 1.17)	<0.001*
BMI	1.04 (1.03 - 1.12)	<0.001*
Physical activity	0.98 (0.96 - 0.99)	0.001*

DM risk in the FinnGen cohort

The MR analysis in the FinnGen consortium further explored the potential causal associations between genetically estimated lifestyle factors and the risk of developing DM. Exploring dietary patterns, a considerable 71% reduction in the risk of developing DM was associated with a genetically estimated higher intake of fruits (OR=0.29, 95% CI: 0.15-0.53, p<0.001). Similarly, higher cheese consumption was also associated with a substantial 52% decrease in DM risk (OR=0.48, 95% CI: 0.31-0.73, p<0.001). However, in contrast to the UKB findings, genetically estimated coffee consumption was associated with a striking 3.4-fold increased risk of DM (OR=3.40, 95% CI: 1.64- 4.02, p = 0.001).

Evaluating smoking habits, smoking initiation displayed an 18% increase in DM risk (OR=1.18, 95% CI: 1.10 - 1.28, p < 0.001), while smoking intensity exhibited no statistically significant association (OR=1.04, 95% CI: 0.96 - 1.12, p = 0.328). Maternal smoking was found to significantly elevate offspring DM risk by 168% (OR=2.68, 95% CI: 1.21 - 3.40, p = 0.013).

**Figure 3:**
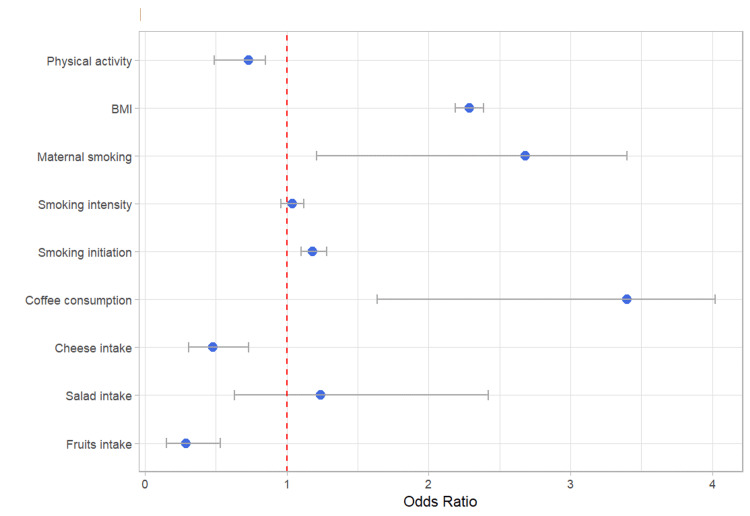
Dietary habits and lifestyle impact on diabetes mellitus risk: FinnGen cohort BMI: body mass index; FinnGen: Finnish health research environment for genomic research

For obesity, a higher BMI demonstrated a considerable effect, associated with a remarkable increase in DM risk per one standard deviation higher BMI (OR=2.29, 95% CI: 2.19 - 2.39, p < 0.001). Finally, greater physical activity levels were significantly associated with a 27% reduction in DM risk (OR=0.73, 95% CI: 0.49 - 0.85, p=0.009) (Table 3, Figure 3).

**Table 3:** Overview of DM findings in the FinnGen cohort BMI: body mass index; CI: confidence interval; The asterisk indicates statistically significant values at p < 0.05 (*: P<0.05)

Risk factor	OR (95% CI)	P value
Fruits intake	0.29 (0.15 - 0.53)	<0.001*
Salad intake	1.24 (0.63 - 2.42)	0.461
Cheese intake	0.48 (0.31 - 0.73)	<0.001*
Coffee consumption	3.40 (1.64- 4.02)	0.001*
Smoking initiation	1.18 (1.10 - 1.28)	<0.001*
Smoking intensity	1.04 (0.96 - 1.12)	0.328
Maternal smoking	2.68 (1.21 - 3.40)	0.013*
BMI	2.29 (2.19 - 2.39)	<0.001*
Physical activity	0.73 (0.49 - 0.85)	0.009*

## Discussion

This two-sample MR study provides novel evidence strengthening causal associations between modifiable lifestyle factors and the risk of developing DM. Most of the observed causal associations aligned with prior MR studies. We were able to reinforce these previous findings in our current MR analysis through the use of a much larger dataset for DM and expanded numbers of genetic variants as instrumental variables for the exposures of interest. Our findings explored the associations between diet, smoking, physical activity, obesity, and DM outcomes.

Regarding dietary patterns, our results demonstrate an 8-71% reduction in DM risk with higher genetically predicted fruit intake, which aligns with findings from meta-analyses showing a lowered DM risk with higher fruit consumption [30,31]. The fiber, antioxidants, and phytochemicals in fruits appear to act through complementary mechanisms regulating blood glucose, inflammation, and body weight to improve pathways related to diabetes risk [30-32]. However, some studies indicate specifically insoluble cereal fibers, rather than soluble fibers from fruits, show more consistent associations with reduced diabetes risk [33]. This discrepancy may reflect limitations in using a small number of SNPs in our genetic instrument for total fruit intake. Additionally, other components of fruits beyond fiber likely contribute to risk reduction through pathways not isolated in observational fiber studies [34, 35]. While soluble fiber alone may not be strongly protective, fruits likely play a role in diabetes prevention as part of an overall high-quality diet. Further research is needed to elucidate the mechanisms and reconcile these discordant findings. Our analysis provides initial evidence suggesting a causal protective effect of higher fruit intake against diabetes development.

Similarly, the 4-52% lowered DM risk seen with higher genetically instrumented cheese intake reinforces observational findings of reductions in diabetes risk with greater cheese consumption [3,4]. Dairy products contain various nutrients and bioactive components that may improve insulin sensitivity and secretion through several interconnected mechanisms to lower diabetes risk. Dairy proteins like whey and casein can stimulate insulin release and enhance insulinotropic responses [36]. Dairy's high calcium and magnesium content optimizes pancreatic beta-cell function and insulin signaling pathways [37, 38]. Vitamin D in fortified dairy may improve insulin receptor expression and have anti-inflammatory effects [39]. Probiotics in fermented dairy help regulate gut microbiota and are favorable for glucose metabolism [40]. While the role of dairy fat remains unclear, the collective effects of dairy's nutritional profile appear to act through pathways improving glycemic control and insulin sensitivity to reduce diabetes risk. Further research is warranted to better elucidate the mechanisms, but the nutrient composition of dairy foods may favorably influence glucose homeostasis [41,42]. Our MR results validate recommendations to increase the intake of dairy products to prevent diabetes.

Our contradictory findings for coffee's effect on diabetes risk align with the mixed results in prior literature and underscore the difficulties in isolating the specific impact of coffee consumption [43,44]. The striking 3.4-fold increased diabetes risk with heavier coffee drinking in FinnGen must be interpreted with caution, given potential limitations. First, confounding remains possible if coffee drinkers have other lifestyle habits affecting diabetes risk. For instance, coffee is often consumed with added sugar, and higher intakes of sugar-sweetened coffee may increase diabetes risk [45,46]. Our genetic instruments cannot account for these types of behavioral confounding factors. Secondly, we utilized only a small number of SNPs as proxy measures for coffee consumption. The limited number of genetic variants may provide less precise and potentially distorted estimates of the effect of coffee intake itself. The disproportionately large effect observed based on few SNPs could reflect an unreliable instrumental variable that requires a more robust genetic instrument with greater numbers of coffee-related polymorphisms.

In summary, while caffeine may acutely impair glucose metabolism at high doses [47], our MR analysis does not provide convincing evidence that coffee consumption specifically has a causal effect on diabetes development. The reliability of the findings is questionable due to confounding factors and limitations in the genetic instruments for coffee intake. More rigorous studies are needed to elucidate the relationship between coffee, caffeine, and diabetes risk.

Our analysis provides robust evidence that higher BMI substantially increases the risk of developing DM. Each standard deviation increase in BMI was associated with 4% and 129% greater diabetes odds in the UKB and FinnGen cohorts, respectively. These results reinforce the well-established bidirectionally causal relationship between obesity and diabetes, which has been documented across numerous epidemiologic studies and trials [48,49]. The pathophysiologic mechanisms linking obesity to diabetes are multifaceted. Excess adiposity, particularly accumulated visceral fat, induces chronic low-grade inflammation and altered adipokine secretions that contribute to insulin resistance and impaired insulin signaling [50,51]. Ectopic fat deposition in the liver and skeletal muscle by triglyceride accumulation also interferes with insulin action in those metabolic tissues. Weight gain further stimulates compensatory hyperinsulinemia in an attempt to overcome insulin resistance and maintain glucose homeostasis [52]. However, sustained insulin resistance eventually leads to pancreatic beta cell exhaustion and dysfunction, hastening the progression to diabetes [53]. Simultaneously, diabetes can predispose individuals to additional weight gain, further exacerbating the condition. Persistent hyperglycemia promotes compensatory hyperinsulinemia, which can upregulate hormones, increasing appetite and caloric intake [54]. This sets up a deleterious cyclical feedback loop where excess weight promotes diabetes onset and progression, and the diabetic state reciprocally exacerbates adiposity.

Breaking this vicious cycle between obesity and diabetes requires integrated interventions targeting weight management, glycemic control, and insulin sensitivity. Our study further validates lifestyle and behavioral modifications focusing on diet, exercise, and maintaining healthy body weight as first-line measures for diabetes prevention and care. Weight loss and activity combat insulin resistance and fat deposition while managing hyperglycemia prevents the exacerbation of obesity in a reciprocal fashion. A multifaceted approach is imperative to attenuate the reinforcing bidirectional relationship between excess adiposity and diabetes [55,56].

In both cohorts, genetically predicted higher levels of physical activity were associated with significantly lowered diabetes risk by 2% in UKB and 27% in FinnGen. This reinforces extensive literature demonstrating robust protective effects of exercise and activity on diabetes development and outcomes [57,58]. Physical activity enhances skeletal muscle glucose uptake through increased GLUT4 translocation, insulin signaling, capillary density, and mitochondrial function. Muscle contraction also releases myokines, improving systemic insulin sensitivity [59]. However, DM complications like neuropathy and risk of injury or hypoglycemia can reduce activity, worsening control [60]. Diabetes and inactivity form a vicious cycle requiring lifestyle promotion, glucose monitoring, nutrition therapy, and tailored exercise to improve DM outcomes. This MR study highlights physical activity as a key protective factor against DM, mediated through impacts on skeletal muscle. However, diabetes control also enables patients to exercise safely. An integrative approach combining activity promotion, glucose management, and personalized exercise prescriptions can break the cycle between diabetes and inactivity. Our findings reinforce physical activity as a critical prevention and management strategy for combating the diabetes epidemic.

While a more complex picture emerged for smoking intensity, our study provides consistent evidence that smoking initiation and maternal smoking during pregnancy increase subsequent diabetes risk. Smoking initiation elevated diabetes odds by 18% in FinnGen and 1% in UKB. Remarkably, maternal smoking increased diabetes odds in offspring by 13% (UKB) to 268% (FinnGen). These findings reinforce prior studies demonstrating smoking is an independent and potentially modifiable risk factor for diabetes development [61]. Proposed explanations include inflammation, oxidative stress, and epigenetic changes induced by cigarette smoke components like nicotine. Maternal smoking may also impair pancreatic development or cause epigenetic modifications in offspring [62]. However, other research has paradoxically shown maternal smoking may reduce type 1 diabetes risk in offspring [63,64]. The mechanisms underlying this discrepancy remain unclear. Overall, our MR analysis provides strong evidence to guide public health measures focused on controlling tobacco use, especially among vulnerable subpopulations like pregnant women, to alleviate the smoking-related diabetes burden. Integrating smoking cessation treatment into diabetes education and care may further help patients with diabetes quit. While the dose-response relationship requires further elucidation, the unequivocal risks of smoking initiation underscore the need for primary prevention efforts.

The current investigation exploring the causal association between modifiable exposures and diabetes mellitus has some limitations to be considered. The study utilized the UKB and FinnGen datasets, which, while large in scale, might not be generalizable to other populations. Additionally, the analysis focused on genetic predispositions to certain diets and clinical characteristics. Other potential risk factors for DM, such as stress, were not examined. Though MR is a robust technique for detecting causal relationships, it still relies on assumptions like the lack of pleiotropic effects of genetic variants. Moreover, this study did not explore the potential interactions between studied risk factors and their combined influence on diabetes risk. Further research with diverse populations, more exhaustive risk factor analyses, and evaluation of gene-environment interplay would build on these important findings.

## Conclusions

This two-sample Mendelian randomization analysis detected the causal effects of several modifiable exposures on the risk of developing diabetes. Genetically predicted higher BMI, smoking initiation, and maternal smoking showed causal effects elevating diabetes risk. Conversely, factors consistently associated with reduced diabetes risk were higher genetically predicted fruit intake, cheese consumption, and physical activity levels. While further research is required to elucidate the mechanisms fully, this investigation provides valuable evidence on potentially modifiable causal factors implicated in diabetes pathogenesis.
